# Exercise Augments the Effect of SGLT2 Inhibitor Dapagliflozin on Experimentally Induced Diabetic Cardiomyopathy, Possible Underlying Mechanisms

**DOI:** 10.3390/metabo12070635

**Published:** 2022-07-11

**Authors:** Mamdouh Eldesoqui, Zienab Helmy Eldken, Sally Abdallah Mostafa, Rasha Hamed Al-Serwi, Mohamed El-Sherbiny, Nehal Elsherbiny, Zuhair M. Mohammedsaleh, Noha Hammad Sakr

**Affiliations:** 1 Department of Anatomy, Faculty of Medicine, Mansoura University, Mansoura 35516, Egypt; mamdouhbasheir@gmail.com; 2 Department of Physiology, Faculty of Medicine, Mansoura University, Mansoura 35516, Egypt; zienabeldken@yahoo.com; 3 Department of Medical Biochemistry and Molecular Biology, Faculty of Medicine, Mansoura University, Mansoura 35516, Egypt; sallyabdallah@mans.edu.eg; 4 Department of Basic Dental Sciences, College of Dentistry, Princess Nourah bint Abdulrahman University, Riyadh 11671, Saudi Arabia; rhalserwi@pnu.edu.sa; 5 Department of Basic Medical Sciences, College of Medicine, AlMaarefa University, Riyadh 11597, Saudi Arabia; msharbini@mcst.edu.sa; 6 Department of Pharmaceutical Chemistry, Faculty of Pharmacy, University of Tabuk, Tabuk 71491, Saudi Arabia; 7 Department of Biochemistry, Faculty of Pharmacy, Mansoura University, Mansoura 35516, Egypt; 8 Department of Medical Laboratory Technology, Faculty of Applied Medical Sciences, University of Tabuk, Tabuk 71491, Saudi Arabia; zsaleh@ut.edu.sa; 9 Department of Anatomy, Faculty of Medicine, Kafrelsheikh University, Kafr El-Shaikh 33511, Egypt; nohasakr851@gmail.com

**Keywords:** diabetic cardiomyopathy, dapagliflozin, exercise

## Abstract

One of the most prevalent cardiovascular problems linked with type 2 diabetes mellitus (T2DM) is diabetic cardiomyopathy (DCM). DCM is associated with myocardial oxidative stress, inflammation, apoptosis, suppressed autophagy, extracellular matrix remodeling, and fibrosis. The current study aims to investigate the protective effect of sodium-glucose transport 2 inhibitor (SGLT2i) dapagliflozin and/or exercise on DCM. Thirty adult male Sprague Dawley rats are used. T2DM is induced by a 6-week high-fat diet (HFD) followed by a single intraperitoneal (IP) injection of 35 mg/kg streptozotocin (STZ). Rats are divided into five groups, control, diabetic (DM), DM + swimming, DM + dapagliflozin, and DM + dapagliflozin and swimming. Serum glucose, insulin, insulin resistance (HOMA-IR), and cardiac enzymes (CK-MB and lactate dehydrogenase (LDH) are measured. Heart specimens are used for evaluation of cellular oxidative stress markers malondialdehyde (MDA), antioxidant enzymes, glutathione (GSH), and catalase (CAT), as well as mRNA expression of TGF-β, MMP9, IL-1β, and TNF-α. Stained sections with haematoxylin and eosin (H & E) and Masson trichrome are used for histopathological evaluation and detection of fibrosis, respectively. Immunohistochemical staining for apoptosis (caspase-3), and autophagy (LC3) are also carried out. The combinations of SGLT2i and exercise exhibited the most significant cardioprotective effect. It improved diabetic-induced histopathological alterations in the myocardium and attenuated the elevation of serum blood glucose, CK-MB, LDH, myocardial MDA, and mRNA expression of TNF-α, IL-1β, TGF-β, MMP9, and the immune expression of caspase-3. Moreover, this combination increased the serum insulin, myocardial antioxidants GSH and CAT, and increase the immune expression of the LC-3. In conclusion, a combination of SGLT2i and exercise exerted a better antioxidant, anti-inflammatory, and antifibrotic effect in DCM. Moreover, the combination enhances the autophagic capacity of the heart.

## 1. Introduction

Chronic hyperglycemia is the main characteristic of diabetes mellitus (DM), which is caused by decreased insulin action or secretion or both [1]. According to global diabetes prevalence estimates from 2017, 451 million people aged 18–99 years are diabetic, with 693 million people expected to have diabetes by 2045, the majority of whom will have type 2 diabetes [2].

Individuals suffering from DM are susceptible to a significant range of cardiovascular complications [3]. Diabetic cardiomyopathy (DCM) is one of the most common diabetic complications, triggering a high rate of mortality [4]. DCM is characterized by diffuse myocardial fibrosis, hypertrophy, and reduced contractile performance in the absence of ischemia, hypertension, or valvular disease [5].

The development of DCM has numerous pathogeneses, including impaired calcium handling, mitochondrial dysfunction, increased oxidative stress, endothelial dysfunction, and remodeling of the extracellular matrix [6]. Apoptosis is the cornerstone of the development of DCM [7]. Apoptosis may be caused by the direct toxic effect of the metabolic changes in DM such as hyperglycemia and dyslipidemia to the myocardium [8].

Autophagy is a physiological process that occurs frequently in the heart. It promotes proper organelle and protein turnover [9]. A basal level of autophagy is a reaction to cellular stress and is necessary for optimal heart function. Excessive autophagy, on the other hand, can damage proteins and organelles. As a result, autophagy can either increase cell survival or result in cell death [10]. DCM is linked to decreased autophagy and increased apoptosis in the heart. As a result, therapies aimed to enhance the antioxidant capacity, eliminate cellular stress, modulate inflammation, suppress apoptosis, and ameliorate fibrosis may be useful in treating DCM [11].

Sodium-glucose transporter 2 inhibitors (SGLT2i) are antidiabetics that predominantly act on the kidney [12], directly on proximal tubules by blocking the sodium-glucose transporter and reducing the reabsorption of glucose. As a result, the urinary excretion of glucose is increased [13]. Dapagliflozin has proved to be an effective treatment for DCM. Indeed, it has been reported to attenuate myocardial inflammation and oxidative stress, enhance left ventricular function, and ameliorate myocardial inflammasome activation [14]. In this study, we adopted SGLT2 inhibitors as they were superior to other hypoglycemic drugs in cardiovascular complications [15], and we used dapagliflozin as it is a highly selective SGLT2i and it has been reported that it is efficient in the treatment of DM and is used as an effective treatment against type 2 DM and prohibits the development of diabetic nephropathy [16].

Exercise is an effective method of preventing and treating a variety of chronic diseases [17]. It has been reported that cardiovascular diseases can be reduced by habitual and leisure-time exercise [18]. The 2019 American Diabetes Association (ADA) guidelines reported that moderate-to-vigorous intensity exercise for 150 min or more per week is recommended to elucidate protective effects against diabetic complications [19]. Aerobic and resistance training exercises for about 1 h per day for four days per week improved glucose and insulin metabolism and reduced associated cardiovascular complications in T2DM patients [20]. Exercise can delay the onset of diabetic complications or even prevent them. Regular exercise has positive effects on DM and the development of DCM [17]. Although SGLT2i and exercise have been studied in T2DM complications, there is limited data examining the combined effect. Additionally, the effect of combined approaches on the key pathogenic mechanisms of DCM (inflammation, fibrosis, oxidative stress, autophagy, and apoptosis) has not been fully studied. Interestingly, exercise improved the therapeutic outcomes of SGLTi ipragliflozin in patients with T2DM. However, the underlying mechanisms are not fully investigated [21].

Herein, the aim of this work is to investigate the cardioprotective effects of dapagliflozin and exercise on cardiomyopathy in a T2DM experimental model. Additionally, the underlying mechanisms have been elucidated.

## 2. Results

### 2.1. Effect of Swimming and/or SGLT2i on Serum Glucose, Insulin, and HOMA Index

Diabetic rats exhibited a significant increase in blood glucose and HOMA index and a significant decrease in insulin when compared with the control group. The swimming protocol and/or treatment with SGLT2i significantly ameliorate these changes when compared with the DM group, with more significant improvement in the MD + SF group, Table 1.

### 2.2. Effect of Swimming and/or SGLT2i on Cardiac Enzymes

When comparing the diabetic rats to the control, the levels of CK-MB and LDH are significantly higher in the DM group. These levels, on the other hand, were significantly reduced after exercise or/and SGLT2i therapy. Furthermore, SGLT2i with or without swimming was considerably more effective than swimming alone, whereas CK-MB and LDH in the DM + SF group were non-significantly lower than in the DM + F group, Table 2.

### 2.3. Effect of Swimming and/or SGLT2i on MDA, GSH, and CAT

**Figure 1:**
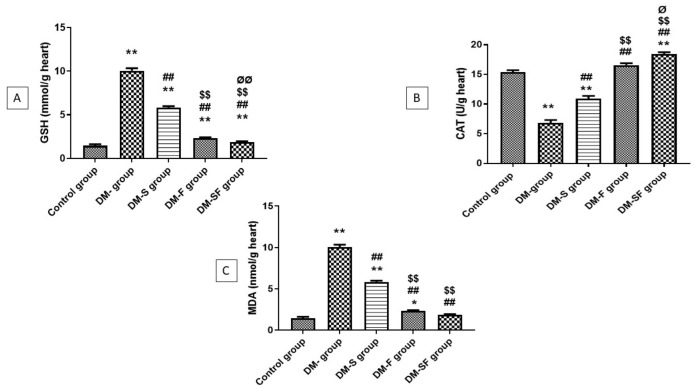
Effect of exercise and/or dapagliflozin on oxidative stress in cardiac tissue of T2DM rats. (**A**) reduced glutathione (GSH), (**B**) Catalase enzyme (CAT), and (**C**) Malondialdehyde (MDA). Data are expressed as mean ± standard deviation. * significance as compared with the control group at *p* < 0.05, ** *p* < 0.01, ## significance as compared with DM group at *p* < 0.01, $$ significance as compared with DM-S group at *p* < 0.01 and Ø significance as compared with DM-F group at *p* < 0.05, ØØ at *p* < 0.01.

There was a significant elevation in the concentration of MDA and a significant reduction in GSH and CAT in the DM group when related to the control group. On the other hand, swimming and/or SGLT2i significantly improved this elevation. There was significant attenuation in MDA in the rats treated with SGLT2i when compared with the swimming group, but a non-significant reduction in the combined SGLT2i and swimming group when compared with rats treated with SGLT2i only. Moreover, SGLT2i significantly increased the level of GSH and CAT than swimming, and the combination of SGLT2i and swimming significantly increase the concentration of GSH and CAT than SGL2i or swimming alone, Figure 1.

### 2.4. Effect of Exercise and/or SGLT2i on TNF-α and IL-1β mRNA Expression in Heart Tissue

**Figure 2:**
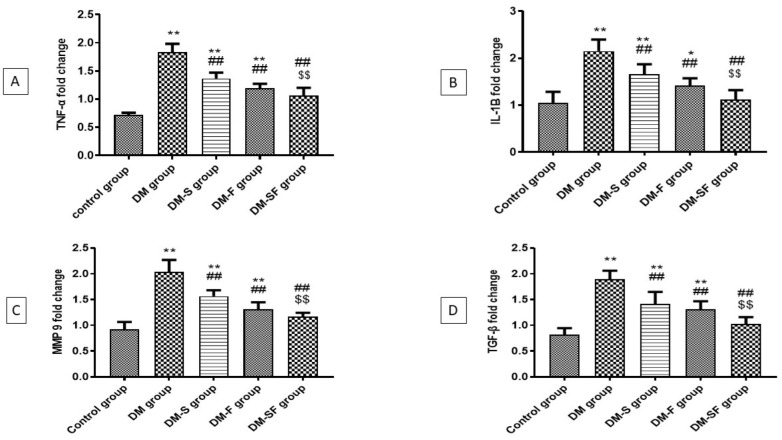
Effect of exercise and/or dapagliflozin on mRNA expression of (**A**) TNFα, (**B**) IL-1B, (**C**) TGF-β, and (**D**) MMP9 in cardiac tissues of T2DM rats. Significance as compared with control group at * *p* < 0.05, ** *p* < 0.01, significance as compared with DM group at ## *p* < 0.01, significance as compared with DM-S group at $$ *p* < 0.01.

The DM group showed a significant elevation in the mRNA expression of inflammatory markers, TNF-α and IL-1β when compared with the control group. Exercise and/or SGLT2i significantly reduced the mRNA expression of TNF-α and IL-1β when compared with the DM group. Treatment with SGLT2i non significantly attenuates the mRNA expression of TNF-α and IL-1β compared to the swimming group, on the other hand, the combination of exercise and SGLT2I non significantly reduced the mRNA expression of TNF-α and IL-1β compared to SGLT2i group and significantly reduced the mRNA expression of TNF-α and IL-1β than exercise group, Figure 2A,B.

### 2.5. Effect of Swimming and/or SGLT2i on MMP9 and TGFβ mRNA Expression in Heart Tissue

The expression of MMP9 and TGF-β mRNA in heart tissue was significantly higher in the diabetic group when compared with the control group. This elevation was significantly ameliorated by swimming or/and SGLT2i. The group treated with SGLT2i showed a non-significant reduction in MMP9 and TGF-β mRNA expression than the swimming group, however, the combination of exercise and SGLT2i significantly attenuated the expression of MMP9 and TGF-β mRNA than exercise alone, Figure 2C,D.

### 2.6. Effect of Exercise and/or SGLT2i on Histopathological Changes in the Myocardium

**Figure 3:**
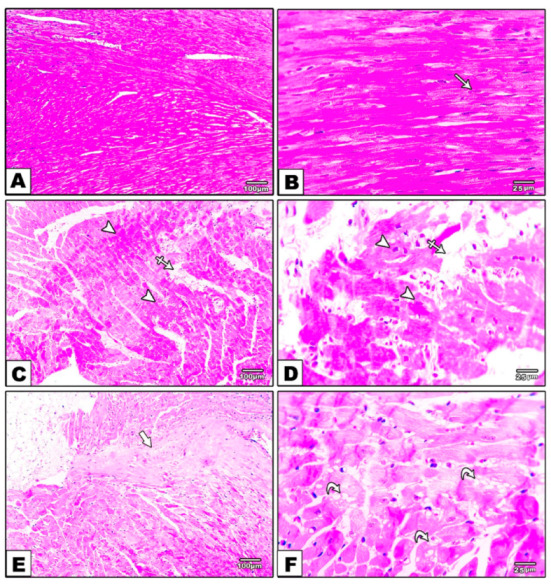

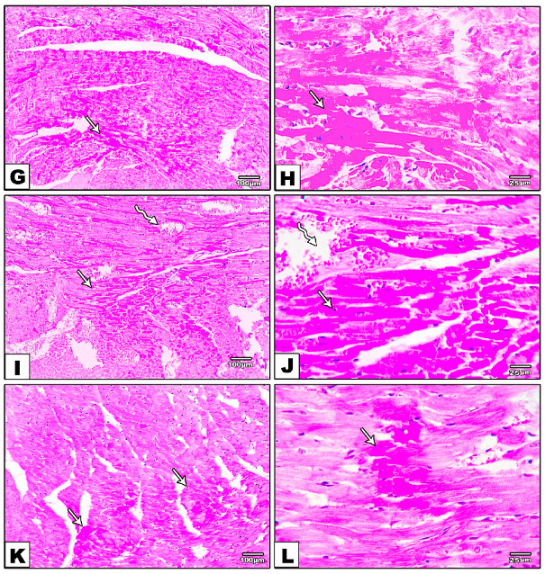
H & E-stained sections, (**A**,**B**) from the control group showing regularly arranged cardiomyocytes and their nuclei (white arrows), (**C**–**F**) from the DM group show multifocal hyalinization areas with pyknotic nuclei (arrowhead), edema (crossed arrow), fibrosis (thick arrow), and vacuolization (curved arrow). (**G**,**H**) from the DM-S group showing a small area of hyalinization with pyknotic nuclei and congestion (white arrow and zigzag arrow), (**I**,**J**) from the DM-F group show a small area of hyalinization with pyknotic nuclei (white arrow) and (**K**,**L**) from DM-SF group showing a regular arrangement of cardiomyocytes and a very minimal small area of hyalinization with pyknotic nuclei (white arrow).

The control group’s heart sections stained with H & E revealed normal myocardial architecture, including regular arrangements of myocardial fibers, cardiomyocytes, and their nuclei. The DM group, on the other hand, had an abnormal arrangement of cardiac fibers, multifocal regions of hyalinization with pyknotic nuclei, edema, fibrosis, congestion, and vacuolization in H & E-stained sections. Furthermore, specimens from the DM-S group had a small region of hyalinization with pyknotic nuclei and congestion, while specimens from the DM-F group had a small area of hyalinization with pyknotic nuclei but no congestion. The heart specimens from the DM-SF group showed a very small area of hyalinization with pyknotic nuclei and a relatively regular arrangement of cardiomyocytes, Figure 3.

**Figure 4:**
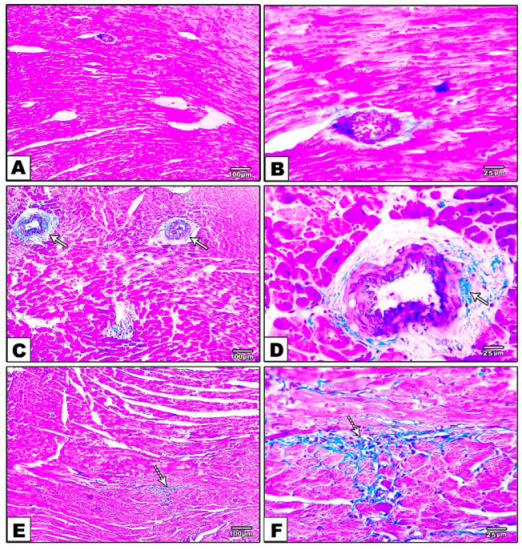

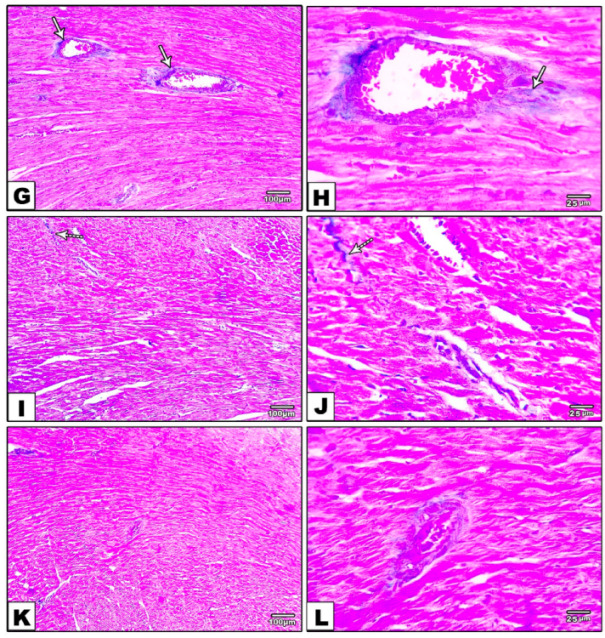
Masson trichrome stained sections. (**A**,**B**) control group showing no fibrosis, (**C**–**F**) DM group showing marked perivascular and interstitial fibrosis and collagen deposition (white arrow), (**G**,**H**) DM-S group showing moderate perivascular fibrosis and collagen deposition (white arrow), (**I**,**J**) DM-F group showing minimal interstitial fibrosis and collagen deposition (white arrow), and (**K**,**L**) DM-SF group showed nearly no fibrosis or collagen deposition.

Histopathological examination of heart sections stained with Masson trichrome showed no collagen deposition in the control group. In contrast, the DM group showed marked perivascular and interstitial fibrosis. Moreover, specimens from the DM-S group exhibited moderate perivascular collagen deposition and fibrosis, while sections from the DM-F group presented minimal interstitial collagen deposition, and the sections from the DM-SF group showed nearly no collagen deposition or fibrosis, Figure 4.

### 2.7. Effect of Exercise or/and SGLT2i on Myocardial Apoptosis

**Figure 5:**
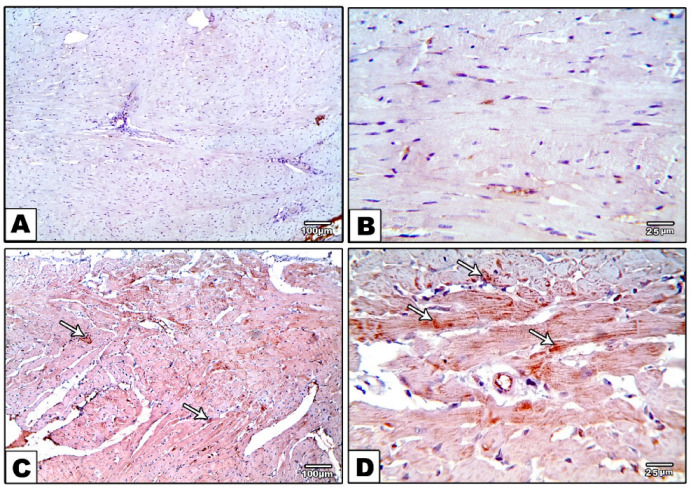

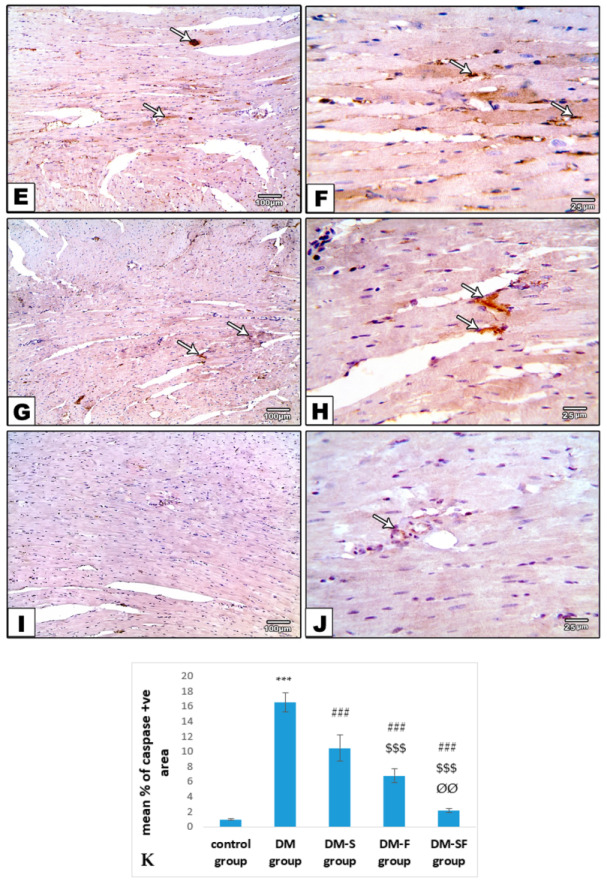
(**A**–**J**) show caspase-3 immunohistochemical staining in different groups. (**A**,**B**) negative cytoplasmic expression of caspase-3 in cardiomyocytes in the control group, (**C**,**D**) significant expression in cardiomyocytes (arrows) in the DM group, (**E**,**F**) moderate of caspase-3 in cardiomyocytes (arrows) in DM-S group, (**G**,**H**) minimal cytoplasmic in cardiomyocytes (arrows) in DM-F group, and (**I**,**J**) very minimal or even no cytoplasmic expression (arrows) in DM-SF group. (**K**) Effect of exercise or/and dapagliflozin on caspase-3 expression as determined by measuring the mean of the percentage of immune-positive area in the studied groups. *** significance as compared with the control group at *p* < 0.001, ### significance as compared with DM group at *p* < 0.001, $$$ significance as compared with DM-S group at *p* < 0.001 and ØØ significance as compared with DM-F group at *p* < 0.01.

The morphometric analysis of the immunoreactive area for caspase-3 showed significant elevation in the diabetic rats when compared with the control group. This elevation was reversed in the DM-S, DM-F, and DM-SF groups. The rats treated with SGLT2i showed significant attenuation of the immunoreactive area for caspase when compared with the exercise group. On the other hand, the combination of swimming and SGLT2i showed a significant decrease in the immunoreactive area for caspase when compared to SGLT2i or swimming only, Figure 5.

### 2.8. Effect of Exercise or/and SGLT2i on Autophagy

**Figure 6:**
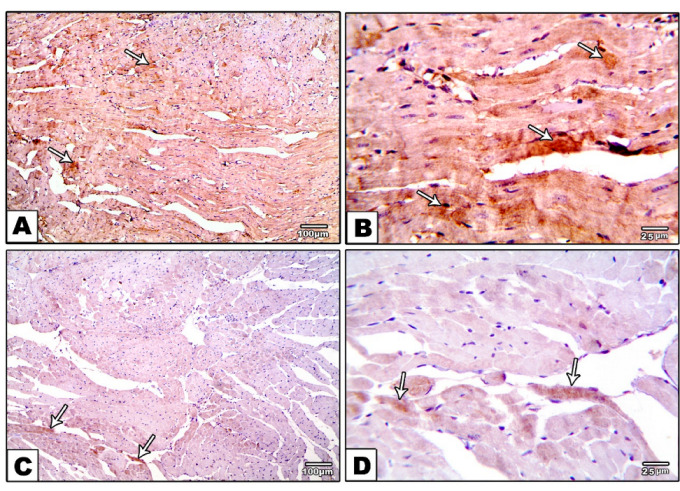

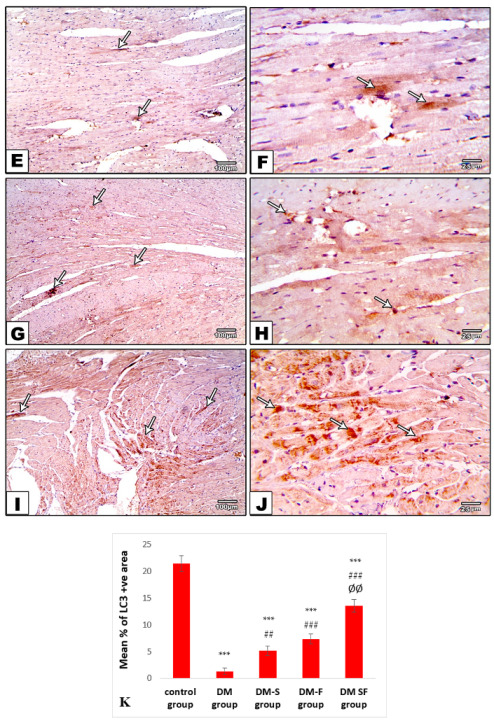
(**A**–**J**) shows the immunohistochemical staining for LC3 in different groups. (**A**,**B**) Strong positive brown reaction in the control group(arrows), (**C**,**D**) in contrast, the positive brown reaction against LC3 decreased in cardiomyocytes in the DM group (arrows), (**E**,**F**) showed mild positive brown reaction (arrows). In the DM-S group, (**G**,**H**) the positive brown reaction was moderately increased (arrows) in the DM-SF group, and (**I**,**J**) the positive brown reaction was markedly increased (arrows) in combined exercise and SGLT2i treatment in DM-SF group. (**K**) Effect of exercise or/and dapagliflozin on the expression of LC3 as determined by measuring the mean of the percentage of immune-positive areas in the studied groups. *** significance as compared with the control group at *p* < 0.001, ## significance as compared with DM group at *p* < 0.01, ### at *p* < 0.001, and ØØ significance as compared with DM-F group at *p* < 0.01.

The morphometric analysis of the immunoreactive area for LC3 showed a significant decrease in diabetic rats when compared with the control group. This result was reversed in the DM-S, DMF, and DM-SF groups. The rats treated with SGLT2i showed a significant increase in the immunoreactive area for LC3 when compared with the exercise group. On the other hand, the combination of swimming and SGLT2i showed significant elevation in the immunoreactive area for LC3 when compared to SGLT2i or swimming alone, Figure 6.

## 3. Discussion

In the present study, the HFD-STZ induced rat model of T2DM was used to evaluate the cardioprotective effect of exercise and dapagliflozin on DCM. HFD-STZ induced rat model of type 2 DM has been extensively used to investigate the effect of various therapeutic agents [22,23]. Hyperglycemia and insulin resistance is owed to the blockage of insulin receptors by the high-fat diet, on the other hand, insulin deficiency could be due to STZ-induced pancreatic injury via the generation of ROS in the pancreatic β-cells, resulting in necrosis [23,24]. Furthermore, a persistent increase in blood glucose may lead to increased production of ROS, accelerating the destruction of the pancreas, especially the β-cells, which have low antioxidant defense capacity [25].

Treatment with SGLT2i or/and exercise significantly attenuates the diabetic effect on glucose homeostasis, including decreased plasma insulin and increased serum glucose, with a more potent effect with the combination of SGLT2i and exercise. Exercise training has been reported to modulate glucose transport into cardiomyocytes under diabetic conditions [26]. In addition, it normalized glucose utilization, increased GLUT4 formation and function, and increased glucose oxidation [27,28]. On the other hand, SGLT2 inhibitors increased glucose and uric acid excretion, demonstrating cardioprotective efficacy [29]. Li et al. [30] reported that fasting and non-fasting blood glucose levels in HFD-induced type 2 diabetes were considerably lower after 8 weeks of empagliflozin treatment.

In the present study, exercise or/and SGLT2i exert a cardioprotective effect as shown by the significant decrease in the level of CK-MB and LDH and attenuation of histopathological alterations including myocardial degeneration and necrosis. The increased serum cardiac biomarkers may be due to disruption of cardiomyocyte membrane integrity and release of cytosolic enzymes in the circulation. A positive association between the levels of cardiac enzymes and the extent of cardiomyocyte degeneration has been described [31,32]. Andreadou et al. [33] reported that empagliflozin (SGLT2i) has a cardioprotective effect against myocardial ischemic injury in animals fed a western diet. Moreover, dapagliflozin reduced cardiomyopathy in type 2 diabetic mice [24]. Moreover, regular exercise training reduced the elevation of cardiac enzymes in diabetic rats [32,34].

The development of DCM is associated with persistent hyperglycemia that leads to myocardial oxidative stress [35]. In T2DM, oxidative stress boosts the production of ROS, leading to myocardial apoptosis [36]. In the current work, diabetic hearts showed enhanced myocardial oxidative stress, similar to results presented in previous studies [37,38]. Treatment with dapagliflozin and/or swimming exercise exerts a cardioprotective antioxidant effect. Both approaches significantly ameliorated oxidative stress and enhanced the antioxidant capacity in the cardiomyocytes. Dapagliflozin had a cardioprotective and antioxidant effect through attenuation of oxidative stress and enhancing the antioxidant capacity of cardiomyocytes [39,40]. The cardiomyocytes express the SGLT1 isoform and there is little evidence for SGLT2 expression [30]. Therefore, Xing et al. [40] using in vitro experiments owed the cardioprotective effect of dapagliflozin to the direct inhibition of ROS production. Additionally, it has been reported that low-intensity exercise decreases oxidative stress and upregulates the expression and function of antioxidant enzymes in cardiomyocytes [41,42]. Exercise enhances glucose metabolism in the diabetic cardiomyocytes and pancreas and enhances anti-oxidative activity in the endothelial cells [43].

Persistent hyperglycemia induces the production of excessive ROS, the release of inflammatory mediators, and DNA damage, leading ultimately to the release of cytochrome C from mitochondria to the cytoplasm and activation of caspase-3 and apoptotic pathways in the myocardium [30,44,45,46]. In the current study, swimming or/and SGLT2i showed a significant reduction of caspase 3 expression in cardiomyocytes. Indeed, SGLT2i administration exhibited a stronger antiapoptotic effect than swimming, and the combination is better than both. Hussein et al. [22] reported that dapagliflozin significantly attenuated caspase 3 expressions and decreased apoptosis in the same model of DCM. SGLT-2 inhibitors attenuated mitochondrial dysfunction, oxidative stress, inflammation, and apoptosis in the heart [47]. In a model of cardiac I/R injury, dapagliflozin increased anti-apoptotic protein (Bcl2) expression, decreased mitochondrial dysfunction, and decreased cardiomyocyte apoptosis [48]. Exercise also exerts a therapeutic antiapoptotic effect as it improves mitochondrial functions [41] and enhances BCL-2 expression in cardiomyocytes of T2DM models [34].

Inflammation is associated with elevated plasma and tissue levels of inflammatory cytokines [49]. NF-κB activation led to an increased level of inflammatory cytokines such as tumor necrosis factor-alpha (TNF-α), which led to cardiac complications in diabetes [50]. Increased levels of TNF α lead to a change of cardiac fibroblast to the inflammatory phenotype that secretes IL1 and IL6, sustaining the inflammation. TNF-α-antagonism ameliorated experimentally induced DCM and was associated with decreased myocardial inflammation and fibrosis [51]. In DCM, myocardial fibrosis is the predominant histopathological change [30,32], characterized by collagen deposition and perivascular and interstitial fibrosis [52]. TGF-β stimulates the formation of the myofibroblasts with excessive production of collagen [53]. MMP9 regulates cardiomyocyte contractility in diabetic hearts, and cardiomyocytes contractile dysfunction is prevented by ablation of MMP9 [54]. In diabetes, there is an elevation of cardiac and plasma MMP9 levels that is accompanied by pathological remodeling [55].

The current work investigated the mRNA expression of the inflammatory cytokines TNFα and IL-1β and reported that it was higher in the cardiac tissues of type 2 diabetic rats. TGFβ and MMP9 expression were also significantly higher, suggesting their activation and participation in DCM. Treatment with SGLT2i or/and swimming significantly attenuates the mRNA overexpression of inflammatory cytokines TNFα and IL-1β and fibrotic markers TGFβ and MMP9. On the other hand, the combination of exercise and SGLT2i is superior to using both separately. Exercise can enhance cardiac remodeling, decrease collagen deposition, and decrease myocardial fibrosis in DCM [34] by lowering blood pressure [28,56]. Moreover, exercise increases matrix metalloproteinase-2 and collagen degradation in obese mice [57]. Moderate exercise significantly attenuated cardiac fibrosis in diabetic hearts [41]. The exercise-induced attenuation of DCM may be attributed to improving energy metabolism, reducing blood glucose, and the deposition of glycogen in the heart [58]. Empagliflozin decreased cardiac oxidative stress and ameliorates myocardial fibrosis. probably due to inhibition of the transforming growth factor β [30], also dapagliflozin decreased blood glucose and blood pressure, improved cardiac function, and attenuated inflammation [12,40]

The current study examined the effect of SGLT2i and/or swimming on autophagy in the diabetic heart. There was a significant reduction in the immunoreactive area of LC3 expression in the heart of the diabetic rats, these findings were in agreement with [59] who stated that autophagy played a pivotal role in cellular homeostasis in cardiac tissues but it is frequently impaired in the diabetic heart. In the present study, treatment with SGLT2i and/or swimming significantly enhances autophagy as it increases the immunoreactive area of LC3 expression when compared with diabetic rats. Experimental autophagy induction has promising effects in the treatment of heart failure [60]. The cardioprotective effects of SGLT2 inhibitors may be due to autophagy stimulation [61]. SGLT2 inhibitors induce autophagy by triggering a fasting or hypoxia-like metabolic state in many tissues, including the heart. This leads to the suppression of oxidative stress and inflammation [62,63], thus enhancing contractile activity and ameliorating the development of cardiomyopathy [62,64,65]. Previous reports have also shown that exercise improved autophagy in cardiomyocytes and prevented cardiac dysfunction [66], thereby restoring normal autophagy function and impairing the progression of cardiovascular complications [67].

## 4. Materials and Methods

### 4.1. Animals

Thirty adult albino male Sprague Dawley rats weighing (160–200 gm) were housed in plastic cages, four animals per cage, under controlled conditions of humidity, and temperature, with free diet and water ad libitum. The rats were kept for two weeks prior to the beginning of the experiment to enable them to acclimatize.

The rats were housed two weeks before the beginning of the experiment to acclimatize. All experimental procedures and techniques were approved by the Kafrelsheikh Faculty of Medicine (MKSU 30-6-21).

### 4.2. Development of Experimental Insulin-Resistant Type 2 DM

As previously mentioned by Elsaid et al. [21], T2DM was induced by a 6-week high-fat diet (HFD) followed by a single intraperitoneal (IP) injection of 35 mg/kg streptozotocin (STZ) freshly produced in cold 0.1 mol/L citrate buffer (pH 4.5). The control rats received the vehicle, 0.1 mol/L citrate buffer (pH 4.5), at a dosage amount of 1 mL/kg, IP. Urine glucose strips were used to check for glucosuria two days later. Blood samples were obtained after another two days to assess serum blood glucose levels. Animals with serum glucose above 200 mg/dL were allowed to continue eating their respective diets until the end of the experiment.

### 4.3. Experimental Design

The rats were divided into the following five equal groups:

- Control group: non-diabetic normal rats that were treated with 0.5 mL saline and 0.1 mol/L citrate buffer (pH 4.5) at 1 mL/kg, i.p. via gastric gavage.
- Diabetes Mellitus (DM) group: rats with T2DM received 0.5 mL saline/day by oral gavage for 6 weeks.
- DM + exercise/swimming (DM-S) group: T2DM rats were subjected to swimming exercise protocol for 6 weeks.
- DM + SGLT2i, Farxiga (DM-F) group: rats with T2DM received SGLT2i (dapagliflozin, FORXIGA, AstraZeneca, Mississauga, ON, Canada, 1 mg/kg/day) via oral gastric gavage for 6 weeks.
- DM + SGLT2i, Farxiga, and exercise, swimming (DM-FS) group: T2DM rats received dapagliflozin treatment and were subjected to swimming exercise protocol for 6 weeks.

### 4.4. Drug Preparation and Administration

Dapagliflozin (Farxiga). Dapagliflozin is commercially available from AstraZeneca (Mississauga, ON, Canada). Dapagliflozin is soluble in sterile water at 0.5 mg/mL. Dapagliflozin was purchased from the local market and used to prepare the dosing solution. Dapagliflozin solution is light-sensitive. Therefore, the stock dosing solution was prepared every week, protected from light, and stored at 4 °C. Dapagliflozin was injected at a dose of 1 mg/kg/day.

### 4.5. Swimming Protocol

For adaptation, rats were immersed in water for one week. The swimming container used was 18 cm in diameter and 30 cm in height, filled with water at 25 °C to 20 cm height. Rats were placed into the container and allowed to swim individually. The duration of swimming was 5 min at the start, and then it increased every day gradually till reaching 15 min/day for 5 days per week for 6 weeks [68].

### 4.6. Blood Sampling and Tissue Collection

At the end of the experiment, rats were anesthetized, and blood was collected by cardiac puncture, left to coagulate, and centrifuged to separate serum to be used for biochemical analysis. Thorax was opened and heart was dissected and divided into three parts, one was preserved in RNA later (10 µL per 1 mg of tissue sample) (Qiagen, Hilden, Germany), kept overnight at 2–8 °C then stored in liquid nitrogen processing, another part was stored in liquid nitrogen for assessment of oxidative stress markers and the last part was fixed in 10% formalin for histopathological and immunohistochemical examination.

### 4.7. Biochemical Study

#### 4.7.1. Measurement of Blood Glucose, Insulin, and Calculation of HOMA Index

Fasting blood glucose (SPIN REACT, Barcelona, Spain) and insulin (Sun-Red biology and technology, Shanghai, China, #cat no 201-11-0708) were assessed in serum using commercially available kits according to the manufacturer’s instructions. Fasting insulin and fasting blood glucose values were used for calculation of the homeostasis model assessment (HOMA) insulin resistance (IR) index using Mathew’s formula [69].

#### 4.7.2. Measurement of Cardiac Enzymes

Creatine kinase (CK-MB, bio-Mérieux Diagnostics, Milan, Italy) and lactate dehydrogenase (LDH, Bayer Diagnostics Ltd., Baroda, India) were measured in serum using commercially available kits according to the manufacturer’s instructions.

### 4.8. Measurement of Myocardial Oxidative Stress Markers

A piece of the left ventricle (about 50 mg) was homogenized in cold buffer and centrifuged at 4000 rpm for 15 min at 4 °C. Then, supernatants were used to measure cardiac tissues content of lipid peroxidation marker, malondialdehyde (MDA), the antioxidant reduced glutathione (GSH) as well as the activity of the antioxidant enzyme catalase by colorimetric method using commercially available kits (Bio-Diagnostics, Dokki, Giza, Egypt) according to the manufacturer’s instructions.

### 4.9. Determination of Tumor Necrosis Factor-α (TNF-α), Interleukin-1B (IL-1β), Matrix Metalloproteinase9 (MMP9), and Tumor Growth Factor-β (TGF-β) Gene Expression by Real-Time PCR (RT-qPCR)

The TRIZOL reagent was used to purify the RNA (Invitrogen, Waltham, MA, USA). Thermo Scientific Maxima First Strand cDNA 161 Synthesis Kit with dsDNase was then used to make complementary DNA (Thermo Fisher Scientific, Rockford, IL, USA). The Primer3PLUS program (v. 0.4.0; 163 http://frodo.wi.mit.edu/ (accessed on 25 December 2021); Table 3) was used to create gene primer sets. According to the method given by Freeman et al., real-time PCR tests were performed using the Applied Biosystem 7500 real-time PCR detection system (Life Technologies, Waltham, MA, USA) [70] with the SensiFAST SYBR Lo-ROX PCR Master Mix Kit (Bioline, London, UK). The entire reaction volume was 20 μL, with the following thermal reaction profile: Denaturation at 95 °C for 2 min, followed by 40 cycles of 95 °C for 5 s, followed by 60 °C for 30 s. The fold induction values were calculated using the Cycle threshold (Ct) method (2^−ΔΔCt^) [71].

### 4.10. Histopathological Examination and Fibrosis Evaluation of the Heart Tissues

Formalin-fixed cardiac specimens were fixed in paraffin, sectioned at 3 µm thickness, and stained with hematoxylin and eosin (H & E) for morphological examination and evaluation of the structural alterations. Other slides were also stained with Masson trichrome to evaluate collagen, extracellular matrix production, and fibrosis.

For both stains, slides were blindly examined using Olympus light microscope, and images were captured using a high-resolution Olympus SC100 digital color camera (Tokyo, Japan) fixed to the microscope.

### 4.11. Immunohistochemical Staining

The tissue sections were deparaffinized and rehydrated. Endogenous peroxidase activity was blocked by treatment with 3% hydrogen peroxide in methanol. The sections were then incubated at room temperature for 15 min in 10% normal rabbit serum in phosphate-buffered saline (PBS) to block non-specific staining. Then, sections were incubated at 4 °C overnight in a moist chamber with the primary rabbit polyclonal anti-LC3 II antibody (diluted at 1:400, cat. no YPA1652, Chongqing Biospes Co., Chongqing, China) for staining of LC3 positive cells and the primary rabbit polyclonal anti-active Caspase 3 antibody (diluted at 1:800, cat. No GB11532, Servicebio Co., Wuhan, China) for staining of Caspase 3 positive cells. After washing with PBS, the tissue sections were incubated with Diaminobenzidine/peroxidase substrate diaminobenzidine tetrahydrochloride (DAB) substrate to produce a brown-colored signal. The sections were processed for a counterstain with hematoxylin. Negative controls underwent all the steps except for exposure to the primary antibody.

### 4.12. Morphometric Analysis of Immunohistochemical Results

The morphometric analysis was carried out using the NIH Image J program (National Institute of Health, Bethesda, MD, USA) [72]. Images were taken with an Olympus^®^ SC100 digital camera and a light microscope. The percentage of area occupied by brown pixels in the analyzed fields was used to calculate the positive expression of Caspase-3 and LC3 (×400). The data were reported as mean SD.

### 4.13. Statistical Analysis and Data Interpretation

Data were analyzed using IBM SPSS Statistics for Windows, and GraphPad Prism 8.0.2. Numbers and percentages were used to describe qualitative data. After confirming normality with the Shapiro-Wilk test, quantitative data were presented using mean and standard deviation for parametric data. The acquired results were judged significant at the (0.05) level. The post hoc Tukey test was used for pair-wise comparisons after the one-way ANOVA test was used to compare more than two independent groups.

## 5. Conclusions

Exercise improves the effect of dapagliflozin on DCM. The combination of dapagliflozin and exercise ameliorates glucose homeostasis, attenuates myocardial oxidative stress, and enhances the antioxidant capacity of the cardiomyocytes. Moreover, this combination has a strong anti-inflammatory and antiapoptotic effect. The combination also attenuates myocardial fibrosis, controls the extracellular matrix remodeling, and restores the capacity for autophagy in the cardiomyocytes of T2DM rats. However, the effect of dapagliflozin on SGLT2 expression in cardiac tissues in DCM warrants further investigation.

## Figures and Tables

**Table 1:** Effect of exercise and dapagliflozin on glucose homeostasis in different groups data presented as mean ± standard deviation.

	Control Group	DM Group	DM-S	DM-F	DM + SF
Blood glucose (mg/dL)	96.00 ± 8.60	383.33 ± 17.79 **	186.17 ± 9.24 **^,##^	152.83 ± 9.91 **^,##,$$^	131.50 ± 3.51 **^,##,$$,ØØ^
Insulin (U/mL)	11.56 ± 0.941	6.82 ± 0.314 **	8.13 ± 0.403 **^,##^	9.58 ± 0.421 **^,##,$$^	10.90 ± 0.387 **^,##,$$,Ø^
HOMA-IR	2.72 ± 0.316	6.44 ± 0.250 **	3.72 ± 0.079 **^,##^	3.45 ± 0.464 **^,##,$$^	3.53 ± 0.058 **^,##,$$,Ø^

** significance as compared with the control group at *p* < 0.01, ^##^ significance as compared with DM group at *p* < 0.01, ^$$^ significance as compared with DM-S group at *p* < 0.01 and ^Ø^ significance as compared with DM-F group at *p* < 0.05, ^ØØ^ at *p* < 0.01.

**Table 2:** Effect of exercise and dapagliflozin on cardiac enzymes data presented as mean ± standard deviation.

	Control Group	DM Group	DM-S	DM-F	MD + SF
CK-MB (U/L)	20.13 ± 1.50	52.08 ± 4.63 **	36.23 ± 2.39 **^,##^	26.46 ± 2.23 **^,##,$$^	23.55 ± 1.53 *^,##,$$^
LDH (U/L)	251.00 ± 7.54	965.83 ± 64.99 **	519.67 ± 7.11 **^,##^	305.00 ± 11.83 *^,##,$$^	276.83 ± 6.55 ^##,$$^

* significance as compared with the control group at *p* < 0.05, ** at *p* < 0.01, ^##^ significance as compared with DM group at *p* < 0.01, ^$$^ significance as compared with DM-S group at *p* < 0.01.

**Table 3:** Sequence of primers used in RT-qPCR.

Gene	Forward Primer	Reverse Primer	Product Length	Reference Sequence
IL-1β	GCTATGGCAACTGTCCCTGA	CATCTGGACAGCCCAAGTCA	136	NM_031512.2
TNF-α	GGCGTGTTCATCCGTTCTCT	CCCAGAGCCACAATTCCCTT	133	NM_012675.3
MMP9	TGGGCATTAGGGACAGAGGA	TTTCCCCTGTGAGTGGGTTG	139	NM_031055.2
TGF-β	CTTTGTACAACAGCACCCGC	CGGGTGACTTCTTTGGCGTA	94	NM_021578.2

## Data Availability

The data presented in this study are available on request from the corresponding author. The data are not publicly available due to privacy.
